# Microbial Metabolomics

**DOI:** 10.2174/138920211797248619

**Published:** 2011-09

**Authors:** Jane Tang

**Affiliations:** Center for National Security and Intelligence, Noblis, Falls Church, Virginia, USA

**Keywords:** Metabolomics, metabolome, microbiota, microbiome, systems biology.

## Abstract

Microbial metabolomics constitutes an integrated component of systems biology. By studying the complete set of metabolites within a microorganism and monitoring the global outcome of interactions between its development processes and the environment, metabolomics can potentially provide a more accurate snap shot of the actual physiological state of the cell. Recent advancement of technologies and post-genomic developments enable the study and analysis of metabolome. This unique contribution resulted in many scientific disciplines incorporating metabolomics as one of their “omics” platforms. This review focuses on metabolomics in microorganisms and utilizes selected topics to illustrate its impact on the understanding of systems microbiology.

## INTRODUCTION

This past February marks the 10^th^ anniversary of the unveiling of human genome sequences – a momentous milestone that made headlines around the world [1]. Described as the “crowning achievement in biology”, among the contributions that made this exceptional accomplishment possible was the microbial genome sequencing effort that took place years prior. In 1995, the first complete genome sequences of a free living bacterium, *Haemophilus influenzae,* were published [2]. Since then, new generations of sequencing technologies have allowed genome sequences to be completed from a variety of organisms. At the time of writing this review, genomes from over 1,600 prokaryotes representing different phylogenetic groups in both Domains of the Archaea and the Bacteria have been completed and deposited in Genbank [3]. Moreover, the Joint Genome Institute has released over 500 completed microbial genomes as of July 2011 [4]. 

Ushering in the genomic era witnessed an unprecedented capability to assess cellular information and network interactions, thus expanding knowledge from individual cell to global biological systems. Transcriptomics and proteomics came on the scene shortly after genomics to allow comprehensive cellular assessments at the transcription and translation level. More “omics” followed and in the process a number of high-throughput and powerful analytical methods were developed. These are instrumental in the fruition of metabolomics – an important complement to assess genetic function [5-7]. 

The study of metabolome – the complete set of metabolites produced within an organism – is a reflection of enzymatic pathways and networks encoded within the genome. Additionally, the entire composition of metabolites conveys the interplay of developmental processes and a changing environment over the lifetime of an organism. By monitoring the global outcome of various factors acting on the cell, metabolomics can potentially provide a more accurate snap shot of the actual physiological state of the organism [8-10].

The monitoring of metabolite components in a system (i.e., metabolite profiling) can be traced back to ancient cultures. For incidence, doctors noted the change in patient’s body fluids (e.g., saliva, urine) to diagnose an illness [5, 10]. As analytical technologies became available in recent years, many metabolites were identified as biomarkers for diseases and examples include coronary heart disease, mental disorders, cancer and diabetes [5, 11-13]. Other health-related areas that have capitalized on metabolomics include toxicology, nutrition (nutrigenomics), *in vitro* fertilization and oncology, to name just a few [10, 14-16].

The plant sciences community has also been active in metabolomics research. The high sensitivity of detection afforded by metabolome analyses allowed studies in several areas; such as the differentiation of wild-types from transgenic counterparts in fruits and vegetables, specific chemical detections in medicinal plants, metabolic network reconstructions, as well as compound formation and monitoring in transgenic vegetables [17-21].

The increased interest in metabolomics and continuous broadening of its applications are evident from a surge of publications in this field since 2003 [14]. It is believed that the time has come to initiate this integrated phase of systems biology, bringing together previous and more traditional “bottom-up” approach of gathering cellular information from individual biological organization and its regulation (i.e., genomics, transciptomics, and proteomics) [7]. As the metabolic complement of functional genomics, metabolomics allow a more complete picture because the intermediates of biochemical reactions play a crucial role in connecting different pathways operating in a living cell. Hence, it has a place in systems biology [6, 8, 22]. Collaborations and consortia are already in place to comprehensively assess metabolites, provide repository and produce databases. The Human Metabolome Project and the Consortium for Metabonomic Toxicology (COMET) are prime examples [23, 24]. 

By comparison, metabolomics in microorganisms is a relatively late comer albeit microbial genomics was at the forefront of sequencing technology and prokaryotes lead in the numbers of completed sequenced organisms [25]. Nevertheless, microbial metabolomics is by no means lagging in progress and contribution. This review, therefore, attempts to focus on microbial metabolomics and mentions a few key areas where considerable interest has been generated in the community and significant progress has been made. The subjects are generally categorized as the association of microbial metabolome with humans, with environmental ecosystems, and conclude with metabolomics contribution to metabolic pathway reconstruction and analysis. 

Two terminologies are mentioned in the literature and often interchangeably – metabolomics and metabonomics. According to Jeremy Nicholson who pioneered the latter approach, “the distinction between the two terms is mainly philosophical rather than technical” [10]. Therefore, “metabolomics” is used throughout this review.

## METABOLOMICS AND ITS CHALLENGES

Metabolomics is the study of global metabolite profiles of a cell under a given set of conditions. It is an important component of systems biology that recognizes as a living system, emergent properties cannot be predicted solely by individual parts. Instead, biological outcomes require an integrated approach to study the sum of all systems [22, 26]. As metabolic products reflect the interactions between the cell’s genome and its environment, metabolomics provides unbiased assessment of a cellular state within the context of that particular condition [14, 16]. Since concentrations of intracellular metabolites often reveal aspects of biochemical regulations that are undetectable by other approaches, metabolomics fills in the gaps from the more traditional studies of interactions between genes, proteins and metabolites in individual cells [27]. It also addresses ambiguities resulting from environmental influences on cellular expressions. Additionally, metabolite changes often result in alterations in phenotype and cellular functions which can be followed by analysis of metabolome [6, 16]. Metabolomics has already demonstrated its critical role in bioenergy, environmental interactions, functional genomics and gene discovery, secondary metabolism, genome-wide association mapping, and metabolic modeling in higher organism and microbial systems [5, 7, 14].

The main challenge of metabolomics is largely technical – the ability to identify and quantify the entire set of intracellular and extracellular metabolites with molecular mass lower than 1,000 daltons [6, 28]. The numbers of these compounds vary among different organisms, from hundreds to hundreds of thousands and in many cases their identity maybe unknown. In contrast to genome, transcriptome and proteome analyses, products generated from metabolic reactions are highly variable in their chemical structures and properties [7]. The metabolome may consist of hydrophilic carbohydrates, volatile alcohols, ketones, amino and non-amino organic acids, hydrophobic lipids and complex natural compounds (secondary metabolites such as antibiotics, pigments, non-ribosomal peptides, cofactors) [6, 22, 29]. This makes simultaneously determining the entire set of metabolites at a given physiological state extremely difficult [9, 28]. Further complicating the analyses is the dynamic nature of these metabolites. Constantly in a state of flux, their concentrations and compositions change rapidly in response to environmental stimuli [6, 7, 27]. Correlation of results may not be straightforward, since a direct link between genes and metabolites sometimes does not exist (for example, microorganisms have fewer metabolites than genes) [27, 30]. All these factors contribute to the complexity and difficulty of metabolomics research. 

## RECENT DEVELOPMENTS ENABLING THE STUDY OF METABOLOMICS

Although significant progress has been shown, some researchers consider metabolomics to be still in its infancy or at an emerging stage [5, 27]. Others advocate the time has come to integrate it as one of the important parts of systems biology and to offer it a role complementary to genomics and proteomics [6, 10, 31-33]. Regardless of various opinions, metabolomics has undeniably leveraged on the knowledge and experience gained from other “omics”. Significant developments in three areas have made the study and analyses of metabolome feasible.

### 1 Improvement of Analytical Tools

The first and perhaps foremost outcome in post-genomic era enabling the advancement of metabolomics research is the substantial improvement of analytical tools. This was initially motivated by the need to screen large number of intracellular metabolites in the context of functional genomics. Two technologies commonly associated with metabolome analyses are nuclear magnetic resonance (NMR) spectroscopy and mass spectrometry (MS) [5, 6, 27, 34, 35]. Both methods are capable of handling a wide range of metabolites in a single measurement without the need to pre-select specific analytes. Additionally, these technologies allow the identification of metabolites’ structures and measurement of molecule concentrations [10] As a result, these two technologies are the accepted analytical methods for metabolomics studies in several scientific disciplines [14, 16].

NMR was already in use in the 1940’s, but continued improvements have greatly increased the sensitivity level to enabling the identification of metabolites in biological fluids, plant cells, and microbial cells [8, 10, 15, 17, 23]. Since the procedure does not require physical or chemical treatments, the samples can be recovered afterwards which is an attractive feature of this technique. On the other hand, MS is more sensitive than NMR [6, 35]. There are many variations of MS. The more traditional approach is often coupled to a separation technique, such as chromatography-based methods, to identify and quantify metabolites with good sensitivity and specificity [7, 9, 11, 21, 28, 36]. Newer MS technologies include stand-alone instruments that are capable of separating and detecting metabolites simultaneously. Others utilize surface-based mass analysis with or without matrices, and with ultra-high-accuracy mass analyzers in order to further increase sensitivity, minimize background and reduce sample preparation. Examples mentioned in the literature include direct infusion MS, Matrix-Assisted Laser Desorption Ionization time of flight (MALDI-TOF), Secondary Ion Mass Spectrometry (SIMS), Fourier transform ion cyclotron resonance MS and Orbitrap MS [6, 14, 32, 37]. 

A number of good review articles provide more details on the technical aspects of NMR and MS, compare their advantages and limitations, and discuss some of their unique applications [6, 7, 9, 32, 38]. Therefore, they will not be repeated here. 

### 2 Availability of Genomic Sequences

The last decade witnessed a profound increase in capability to perform low-cost, high-throughput sequencing. From whole-genome shotgun sequencing used in deciphering genetic codes of the first bacterium *Haemophilus influenzae* to the dramatic changes in automation and massively parallel DNA sequencing in recent years, individual genome sequencing is becoming common place [2, 39].

Over 1,000 finished prokaryotic genomes are available in Entrez Genome and many more are near completion. Diverse groups are represented and they include pathogens, extremophiles, endosymbionts, gut commensals and ecologically important members (e.g., nitrogen fixers, carbon fixers) [3]. Some genera are better profiled by the availability of genomes from multiple species (e.g., *Mycobacterium*) and in some cases, genomes of several strains within a species (e.g., *Mycobacterium tuberculosis*). Because next generation and third generation sequencers claim high speed and low cost, producing DNA sequences from microorganisms is no longer confined to large genomic centers. A plethora of sequencing information is now available. This allows comparisons and genome wide analyses to be conducted at all levels. This also enables the assessment of different and unique attributes of an organism within a community as well as detection of its novel metabolic pathways [40-42]. 

Following the sequencing of individual microbial genomes, the high-throughput capability of sequencing technologies makes feasible the next level of systems biology – large scale gene assessment of community members [43, 44]. Most microbes found in nature exist in complex, interdependent communities and cannot be readily isolated and grown in the laboratory. Genomic analyses of whole communities (i.e., environmental genomics) reveal microbial diversity previously unknown. A number of metagenomic projects and consortia from bio-diverse environments have added billions of base pairs to the sequence database [4, 29, 45, 46]. These data are necessary to resolve the intricacies of microbial members residing in different ecological niches and to understand community diversity [41, 42, 47, 48]. The microbial communities associated with humans have also been explored by metagenomic sequencing. For instance, a whole-genome shotgun (WGS) approach was used to define the full genetic diversity and predict gene functions of gut microorganisms [49]. This paves the way for ecosystems biology to combine data generated from metagenomics with those from other meta-“omics” in order to reconstruct system-wide networks in microbial communities [22, 44, 50-52].

### 3 Data Integration, Standards and Models

The organization of the various “omics” in a hierarchical fashion enables an integration approach which becomes the foundation of systems biology [22, 27]. There are multiple benefits but most importantly, integration and correlation of data sets provide insights not obtainable from other techniques [7, 14]. A number of annotation and statistical tools as well as network analysis software and databases have been developed [32, 34, 53-55]. Among them, are those with metabolomics applicability (e.g., Kyoto Encyclopedia of Genes and Genomics, KEGG; Clusters of Orthologous Groups, COG; Entrez Gene, Transport DB and UniProtKB) [22, 29, 31, 50]. A considerable effort has been initiated to establish common interchangeable standards in genomics and proteomics in order to successfully integrate data sets [25, 33]. Furthermore, new models to address specific challenges pose by metabolomics are being developed [7, 14]. These informatics resources are valuable for reconstructing biochemical networks and metabolic engineering in different microbes [22, 27, 32, 50, 55]. 

Because informatics is dynamic, it is likely that adaptable databases and novel analytical tools will continue to arrive on the scene. As more large scale projects take place, they will encounter unique challenges when integrating vast amount of data generated from various platforms [25, 56, 57]. Knowledge gleaned from previous efforts can become the foundation for future endeavors. 

## MICROBIAL METABOLOMICS AND APPLICATIONS

Microorganisms are ideal for conducting systems biology studies because they are easy to manipulate and have crucial roles in human health as well as the biosphere. Microbial metabolomics is one of the platforms for integrating biological information into systems microbiology to facilitate the understanding of microbial interactions and cellular functions.

As a context-dependent subject reflecting the overall physiological state of the cell, the discussion of microbial metabolomics cannot be dissociated from its host or ecological niche. There are a variety of topics and applications reported in the literature. This review focuses on three selected areas to illustrate the recent progress of microbial metabolomics.

### 1 Human Ecosystems 

The Human Microbiome Project (HMP), initiated in 2007, represents a paradigm shift in how microbes are being viewed in human biology. Until recently, bacteria were mostly regarded as intruders that upset the systems which they colonized. Although research findings from different metagenomics projects pointed to the key roles that microbes play, this recognition was mainly confined to environmental microbiology. 

In the last decade, there was an obvious change in the perception of interactions between microbes and humans. After the completion of the Human Genome Project, it was soon realized that microorganisms living inside and on humans (i.e., microbiota) are outnumbered human cells by an estimated factor of ten [29, 56]. If humans are composites of human and microbial cells, the metabolic features of humans are a blend of traits from both [58]. Earlier metagenomics studies supported this idea that the microbiome (i.e., genomes of microbiota), including many taxa that were unknown and uncultured, is crucial for human health, [40, 59-61]. 

As an extension of the Human Genome Project, the HMP provides a great opportunity to integrate complex systems that account for microbial community structure (microbiota), gene content (metagenomics), gene expression (meta-transciptomics and meta-proteomics), and metabolism (meta-metabolomics) [58]. Five sites of the human body were surveyed to provide a comprehensive inventory: the mouth, the gastrointestinal tract, the vagina, the skin and the nasal cavity [56, 58, 62]. Parallel efforts to sequence new reference genomes assist the understanding of the microbial communities. As of the end of 2010, 500 out of the 3,000 relevant microbial genomes have been sequenced [59]. 

Significant findings have already resulted from HMP since its inception. Among them, gut microbiota will be further discussed because this community is perhaps one of the best illustrations for the interaction between human and microbial metabolomes and a number of papers have been published on this topic. This is by no means minimizing the contributions of microbial communities associated with other human colonization sites; in fact, interesting findings are already emerging [60, 62-64]. 

### Gut Microbiota

The microbes (including bacteria, archaea and fungi) residing in the gut have long been known as important components of the intestinal ecosystem [12, 40, 46]. There are approximately 10^14^ microorganisms in the human intestinal tract comprising more than 1,000 species including many that cannot be cultivated [29, 35, 61, 65]. This abundant and diverse population of residents, or microbiota, offers many benefits. These include defense against pathogens, confer immunity, renew gut epithelial cells, harvest inaccessible nutrients, and generate energy by anaerobic metabolism of substrates [35, 40, 58]. With a systems biology approach, host and gut-microbial interaction can be visualized from multi-dimensional perspectives. 

#### a Metabolize Foreign Substances and Toxins

The first report describing the functional attributes of microbiome resulted from a metagenomic analysis of fecal samples from two unrelated, healthy adults [29, 58]. Metabolic function analysis was done by comparing the metagenome to previously-sequenced microbial and human genomes. A number of genes involved in certain metabolic pathways were significantly enriched in the human distal gut microbiome, such as those involved in the metabolism of foreign substances, glycans (plant polysaccharides), amino acids; production of methane (likely as a mechanism to remove H_2_ from bacterial fermentation); and utilization of the MEP (2-methyl-D-erythritol 4-phosphate) pathway to synthesize vitamins and isoprenoids [29].

The contribution of gut microbiota to the metabolism of exotic substances (i.e., xenobiotics) is noteworthy, as most drugs are considered foreign to the body. Analogous to bioremediation, these microbes can detoxify harmful compounds (e.g., carcinogens) which impact the host in a variety of ways. These include susceptibility to cancer, efficiency of drug metabolism, and absorbing derivatives from plants (e.g., flavonoids) [29, 58]. Indeed, a number of reports have indicated differences in gut microbiome content among populations and geographical variability in microbial-related drug metabolism [12, 35]. Furthermore, as host and microbes have co-existed through the centuries, selective pressure resulting in both systems to come up with beneficial enzyme systems [66]. One example is co-metabolism which not only enhances survival by converting potential harmful substances into valuable energy sources, but also expands the metabolic repertoire to transform xenobiotics [35, 67]. 

On the other hand, co-metabolism can have an opposite effect if the breakdown products are harmful to the host. At times, gut residents metabolize toxins or xenobiotics into substances that are potentially more unfavorable, such as carcinogens, with long term consequences [35]. 

Therefore, a complex continuum and dynamic interactions exist between a controlled metabolism of the host and the co-metabolism contributed by both the microbiota and host. The latter is often fed by exogenous sources such as dietary substances and metabolic products formed by gut-microbiota. This results in “combinatorial metabolism” as termed by Nicholson *et al*. and further complicates the measurement and analysis of metabolites as well as their effects on the host [35]. 

#### b Modulate Human Phenotypes

Human phenotypes are affected by microorganisms residing in the gut. Earlier studies indicate human populations can be distinguished by their gut-microbiota which in turn are influenced by a variety of conditions such as lifestyles, history, and physiological makeup [58, 68]. Among the different factors affecting gut residents is diet. The slogan “we are what we eat” has a more profound meaning in light of recent findings – the types of food ingested actually modify the gut microbiome and thus alter host phenotypes [69]. 

An earlier paper by Ley *et al*. described that diet, host morphology, and phylogeny affected the composition of vertebrate gut microbiota [66]. The authors compared human gut microbiome with those from primates and mammals, and followed variations of mammalian gut microorganisms through history as a reflection of their ecological niches and diet changes. They concluded that the shifts of human diet over time influenced human genomes. Two illustrations were provided: an increase in salivary amylase gene correlated with a high starch diet, and lactose intolerance coincided geographically with milk-protein gene diversity in cattle and the locations of cattle-farming sites [66, 70]. The latter example further suggested the changes in human culture, technology, and cooking style have shaped microbial ecology and human microbiome [71]. 

In a more recent report, an association was noted between the number of microbial genes involved in certain metabolic pathways and the host phenotype. In this case, animal studies were conducted to look at obesity. Metagenomic analyses indicated that the microbiome in obese mice had an increased capability to harvest energy from their diets and thus resulted in fat deposition [58, 68]. More specifically, the whole genome shotgun (WGS) approach used to define the full genetic diversity of and functions associated with the gut microbiome showed that gene contents was enriched for those involved in importing and metabolizing otherwise indigestible polysaccharides to short-chain fatty acids. These in turn could be easily absorbed and stored as complex lipids in host adipose tissue. This was further confirmed in germ-free animals by colonizing them with microbiota from obese mice and observing a significantly greater increase in total body fat in comparison to normal mice [49]. 

Like mice, humans harbor two predominant groups of bacteria, namely the Bacterioidetes and the Firmicutes. A similar association of human gut microbiota composition with obesity was noted and the relative proportion of Bacterioidetes was lower in obese people when compared with lean individuals. This ratio changed when obese people were put on a low calorie diet (carbohydrate-restricted or fat-restricted) and lost between 2-6 % of their body weight [68]. These findings demonstrated that the gut-microbiome plays a role in obesity [69]. 

On the other hand, metabolites from the gut microbiota can change in spite of constant diet. This may be due to changes in how food is metabolized (e.g., using different metabolic pathways) or an alteration in gut microbiota resulting from other environmental factors (e.g., stress) [35]. Background variation obviously complicates the interpretation of results generated from host metabolism, microbial metabolome, or a combination of both.

#### c As Etiology for a Disease or Condition

As part of a complex ecosystem, the gut microbiota influences the health status of the host [40, 65, 72]. On the beneficial side, in addition to the aforementioned contributions to the metabolism of various substances, there is evidence that microorganisms promote healthy gut development by forming normal villous structures and renewing gut epithelial cells [35, 58]. Animals lacking a community of microbes in their gut end up with anatomical disorders that often predispose them to disease [67, 73]. 

There is a mutualistic relationship between the host and its gut microbiota. The environment, host genetics and the microbiome all play a part in the homeostasis of the gut [69]. Interactions between the microbiota and the host can influence disease progression [35, 74]. For some, the outcome is positive, resulting in reduced occurrence or controlled disease manifestation. One of the mechanisms is by microbial interactions with epithelial and immune cells in the intestine, such as promoting anti-inflammatory immune response by the indigenous gut bacteria and activating regulatory cells of the host [74]. A recent paper reported that the colonization of germ-free mice with a cocktail of 46 *Clostridium *species was sufficient to induce the accumulation of interleukin-10 (IL-10) cells and T regulatory cells (T_regs_) in the colon [61]. Included in the study was a mouse model of colitis that resembled human irritable bowel syndrome, and the symptoms were significantly suppressed in mice that were colonized with *Clostridium* species. The authors speculated that a diverse set of metabolites produced by the 46 strains of *Clostridium *contributed to maintaining immune homeostasis in mice and possibly in humans as well [61].

On the other hand, the gut microbial community can exert an adverse effect on hosts, resulting in immune disorders within and outside of the gut. There is evidence that the gut microbiota plays a role in the development and activity of both the innate and the adaptive immune systems [40, 58]. Irritable bowel syndrome (IBS) and Crohn’s disease are perhaps the best known examples of inflammatory intestinal diseases resulting from a dysregulated immune response to the gut microbial community [58]. The former affects 10-20% of world population and evidence indicates that intestinal fermentation patterns reflective of microbial metabolism were changed in these patients [75]. This was confirmed by a genomic study showing significant alterations in microbial composition in IBS patients when compared to healthy individuals [65]. Similarly, metagenomic analysis showed a reduced complexity of Firmicutes in fecal samples from Crohn’s disease patients. The change of bacterial diversity has several consequences. First, the reduced proportion is likely compensated for by an increased representation of Gram negative bacteria known to express more proinflammatory molecules (such as lipopolysaccharides). Second, major groups of Firmicutes in the gut produce large amounts of butyrate, a metabolite that serves as an anti-inflammatory substance and inhibits cytokine mRNA expression in the mucosa [76]. Lastly, the loss of butyrate producers likely upsets the interaction between epithelial cells and gut microbe. This then contributes to the development of Crohn’s disease-associated ulcerations as well as other dysbiosis-related conditions [77]. 

These are just two examples of gut microbiota acting as etiological agents for gastrointestinal disorders. The aforementioned obesity is one condition which a pronounced change in microbial composition is associated with host pathology. Furthermore, there is indication that the interaction of microorganisms with one of highly expressed mammalian’s receptors in the adipose tissue may predispose the host towards type II diabetes, morbid obesity and heart disease [35]. The disruption of microbiota, such as caused by antibiotic treatment, can lead to dysregulation of host immune system and increase susceptibility to disease [76]. One notable example is antibiotic-associated diarrhea caused by the proliferation of *Clostridium difficile *resulting in pseudomembranous colitis, a health concern especially in hospitals and nursing home facilities [78, 79]. Additional abnormalities associated with gut microbiota have been demonstrated in mouse models; these include allergies, arthritis and central nervous system inflammation [74]. 

#### d Applications of Gut Microbiome

As the HMP proceeds to unravel more features of the intestinal microbiota and their implications, different applications to human and animal health are likely continuing to abound. One particular area that has gained considerable momentum is probiotics, which illustrates that indigenous species can be manipulated (in this case, by ingesting adequate amounts of beneficial bacteria) to confer health benefits on the host. This is based on the notion that probiotic organisms possess the ability to transiently colonize the gastrointestinal tract to allow a balance in gut microbiota. Mechanisms of action were deduced by clinical trials and they include strengthening and maintaining the intestinal barrier, modulating immune responses, enhancing microbial flora and resisting colonization by pathogens [40, 80]. Furthermore, the metabolic products of certain probiotic bacteria may exert an antagonistic effect on pathogens by lowering pH and/or secreting antibacterial substances. Another proposed protective mechanism is the exclusion of pathogens from mucosal binding sites and nutrients by competing probiotic organisms, thus preventing their establishment in limited niches [61, 79]. 

Many microbes have been considered and tested for their probiotic ability. In general, species of *Lactobacillus* and *Bifidobacterium* are model organisms frequently utilized to show efficacy [72, 78, 79]. The recent advancement in genomics provides further insights into how probiotic bacteria react to the gastrointestinal tract environment. In fact, probiogenomics is joining systems biology to perform large scale analysis of probiotic bacterial genomes and to elucidate molecular basis of probiosis [40, 72]. 

The fact that individuals are affected by their gut microbiome in response to drugs has implications for personalized medicine and devising new approaches to drug discovery. In the future, gut microbiome determination may be considered along with Single Nucleotide Polymorphisms (SNPs) assessment in an individual for negative effects from drug metabolism (such as adverse drug interaction) [35]. The Consortium on Metabonomic Toxicology (COMET) has already considered this aspect as one of its objectives [10, 23]. 

### 2 Environmental Ecosystems 

Microbial communities are an essential component of ecosystems. Until recently, the understanding of these communities was largely restricted to microorganisms that could be cultured [42]. The development of DNA sequencing technologies and novel approaches revolutionized the field of environmental microbiology, allowing whole communities, including uncultivated members, to be studied [41, 45, 47, 51, 81]. 

The microbial ecosystem encompasses all microorganisms and their neighbors (including higher organisms such as plants and animals) living in a particular niche. The system is dynamic with various factors affecting the niche; these include environmental conditions (e.g., temperature, moisture), chemical compositions (e.g., acidity, nutrient concentrations) and structure of the surroundings (e.g., solid, fluid). Therefore, the components and interactions within the community range from simple (one or two species in a well-controlled growth condition) to complex (e.g., soil, ocean, rhizosphere, waste water) [42, 44, 50, 52]. Interactions between residents are generally categorized as symbiotic, mutualistic or competitive. 

Among the dynamics that result in a defined outcome are metabolic activities from each member. As seen in human gut microbiota, the community population and interactions have pronounced effects on the host as well as each other [58, 68, 77]. Thus, metabolomics plays a crucial part in understanding the populations and interactions within the microbial ecosystems. It leverages results and developments from the other “omics” and further provides insights into community functions [50, 52, 82]. Three topics are selected as illustrations. 

#### a Biodiversity

Before global diversity became a Millennium Development Goal in the early 2000’s, microbiologists were already aware that prokaryotes constitute the unseen majority [25, 83]. Metagenomic projects from different environments confirmed the vast number of microbes on earth because population assessment is no longer impeded by the ability to culture them [4, 42, 48]. Large scale technologies in systems biology are expanding into microbial ecosystems aiming at understanding community functions [36, 50].

The contribution of metagenomics to microbial community is indisputable, especially in diversity assessments that included uncultivated Archaea [84]. As highly complex communities (e.g., soil, ocean) are being explored, the limitations of metagenomic sequencing were soon realized (such as assembling numerically dominant species and insufficient coverage to name a few) [25, 42, 48, 85]. Metagenomic sequencing can still serve as a hypothesis-generating tool; however, functional diversity stemming from microbial diversity encompasses a wide range of metabolic activities. Therefore, new approaches are necessary to circumvent the limitations posed by metagenomic analysis. This is especially critical when assessing uncultured members of the community [50, 84]. 

Recent progress has been made in this respect. One paper reported utilizing fragmented genomic data to predict the encoded proteins, instead of relying on the types of microbes that produce them, to determine functions and features of the community [42]. When operons from several different types of environmental samples were analyzed, results indicated correlations with specific environmental conditions (soil versus ocean) which were reflective of metabolic demands. Thus, predicted meta-proteomics from genomic data paved the way for functional studies in microbial community [42]. 

To address metabolic capabilities and ecological functions among the uncultured members of the community, Stepanauskas and Sieracki devised an alternative to metagenomic sequencing [85]. Instead of using environmental DNA extracts, the authors sequenced multiple genes from individual bacterial cells and assess these genes in members of the community. This approach was shown to be more productive in metabolic mapping of uncultured microorganisms because their metabolic genes could be detected directly. Specific examples included genes involved in significant biogeochemical functions in marine environment such as photometabolic systems, nitrogen fixation and nitrate utilization. The results also proposed major carriers of certain metabolic pathways in this marine ecosystem [85]. 

#### b Metabolic Cooperation

Microorganisms interact in different ways to benefit each other within a community. Metabolic cooperation is one of them, and it is achieved by synergistic relationship or mutual exchange of metabolites [50]. The former is a simpler association and involves two microorganisms (i.e., co-culture) transferring intermediate metabolites from one member to another. For example, *Acetobacterium woodii* and *Methanosarcina barkeri* interact cooperatively to degrade glucose *via *acetate and form methane as the end product. This type of synergistic relationship, or syntrophic association, is beneficial because it is energy-efficient and allows maximum utilization of available resources [86]. 

Mutual exchange of metabolites is another cooperative approach and it can occur in biogeochemical cycling of nutrients and elements or breaking down of complex polymers by multiple organisms. During these processes, different members participate in complementary pathways and the metabolites generated are being transported in and out of the cell. The community structures are often complicated and the populations vary over time [50].

Co-dependence has also been noted among members of the human microbiota. Different metabolite production and utilization pathways take place to provide nutrients or secondary metabolites for energy. Other members remove waste products by metabolizing them as energy sources and thus prevent toxic build up. These metabolic interactions contribute to homeostasis in the intestinal system [76]. 

Cooperative interactions can be inferred by analyzing the genomic contents of the community. For instance, taxon occurrence patterns in a certain ecological niche could provide insights of metabolic cooperation [50]. As more complete genomes are becoming available, metabolite exchanges between these members can be hypothesized. Furthermore, different computational tools enable metabolic cooperation models to be built from genomic and proteomic data. These objectives are generally accomplished by metagenomics projects.

On the other hand, large scale proteomics can step in if community genomic data is not available. One such example is the reconstruction of community structure and metabolism from a natural biofilm at an acid mine drainage site [36, 82]. As a self-contained environment, a mixture of Bacteria and Archaea carry out biogeochemical activities [81]. The genomes of five predominant members were reconstructed, ranging from near completion to partial recovery of their genomes. Metabolic pathways were deduced from these genomic data in order to understand community interactions and functions. Specifically among the subjects of study were carbon and nitrogen fixation pathways employed by *Leptospirillum* group II and *Ferroplasma* type II, their electron transport chain for respiration, and putative cellulose synthesis for their survival in biofilms. New discoveries were made, and among them were novel cytochromes and light-activated proteins to repair ultraviolet-damaged DNA [82]. 

A follow up study combined metabolomics with high-throughput proteomics to investigate functional differentiation of *Leptospirillum* groups II and III co-inhabiting the biolfilms of an acid mine drainage site[81]. These two species reflect ecological succession, with group II dominating at the earlier phase and group III showing predominance as the biofilm matures. Study results indicated strong metabolomics segregation based on organism type, leading the authors to conclude that evolutionary divergence is associated with the restructuring of cellular metabolic networks, which in turn may lessen competition and allow community members to occupy distinct niches [81]. 

These examples are good illustrations of linking metabolomics with genomics and/or proteomics to assess community functions and interactions. This is possible because acid mine drainage is a low complexity niche dominated by a small number of species with limited genetic exchanges [36, 82].

#### c Cell to Cell Signaling

As microbial communities become more complex, a global systems approach is necessary to understand the formation of consortia, communication between members and functional interaction in a dynamic setting [42, 50]. One type of such interactions between microbes and their environment is cell to cell communication [86, 87]. This is often a cooperation strategy to sense and respond to chemical signals. The best example is quorum sensing which occurs between the same species (i.e., intra-species cooperation) [88]. When cell density is high, bacteria secrete small molecules (or autoducers) to initiate collective behaviors. These include formation of biofilms, bioluminescence, expression of virulence, coordination of enzyme expressions, and establishment of competence for DNA exchange [50, 86-88]. By synchronizing the behaviors of all members in the group, they act like a multi-cellular organism. One of the most studied model organisms for cell to cell communication is the fruiting body formation of a soil bacterium, *Myxobacter xanthum *[86, 87]. Other models for signaling pathways have been reported for pathogens (e.g., *Pseudomonas aeruginosa*, *Vibrio cholerae*), cyanobacteria (e.g., *Anabaena*) and eukaryotes (e.g., *Dictyostelium, Saccharomyces*) [88]. 

Inter-species cooperation is less understood except that interactions are likely to be mutually beneficial. Various systems containing large consortia of different organisms have been found with implications in health care (e.g., biofilms on human teeth, organs) and agriculture (e.g., root nodules of crops) [87, 88]. Communication exists to adjust group behaviors and population densities in order to provide shelter, forage, reproduce and disperse members [86]. 

This brief discussion on cell to cell communication is rather simplistic. In reality, dialogues exist in microbial communities ranging from one- way, two- way to multi- way interactions. Messages are not always friendly but can be mixed, interfering and antagonistic [87]. Currently, reconstruct various inter-species small molecules produced by organisms in a community is rather difficult. It is foreseeable that signaling cascades driving social behavior in environments can be deduced by data generated from components of systems biology, including metabolomics [50]. Data from metagenomics can again spearhead the discovery of novel biosynthetic pathways and from which small molecules exhibiting signaling activity may be inferred [87]. 

#### d Applications

As of July 2011, over 240 metagenomic projects have been completed [4]. The informatics generated from these data sets is invaluable; however, they represent only a component of ecosystems biology. The time has come to combine environmental microbiolomics approach with data from metagenomics, meta-transcriptomics and meta-proteomics to reconstruct microbial ecosystems and understand their parts and connectivity. 

They are many applications for a system-wide approach to assess ecosystems. Among them is utilizing metabolomics to understand the dynamics and interactions of intrinsic bioremediation that takes place in various environments. The Deepwater Horizon blowout in the Gulf of Mexico in 2010 serves as a good illustration for such an opportunity. Scientists were pleasantly surprised to discover the hydrocarbon biodegradation was proceeding faster than expected [89, 90]. Similar to other environmental studies, microbial community composition and structure respond directly to a change in condition; in this case, the change is a dispersed oil plume. Genomics analysis identified γ–Proteobacteria as the dominant deep-sea indigenous microbe responsible for hydrocarbon degradation. This led the authors to speculate that intrinsic bioremediation of oil contaminants probably exists in the deep sea [90]. Approximately three months later, the microbial community changed drastically with members of the previously identified hydrocarbon-oxidizers diminished significantly. Instead, methylotrophs and methanotrophs responded actively to the large scale influx of methane and converting it to CO_2_ [89]. 

These groups of bacteria represent just a small fraction of the deep sea microbial community adapted to this extreme environment. A systems approach to elucidate the dynamic interactions and functions of this unique habitat will have relevance in bioremediation, carbon cycling, metabolic networking and climate change. 

Biogeochemical conversion of organic matter in the ocean is another area that microbial metabolomics can make an impact. Referred to as the biological pump, a series of processes starting with CO_2_ fixation followed by the transfer of organic matter to the ocean resulted in either temporary or permanent carbon storage. Microorganisms are capable of mineralizing both particulate and dissolved organic materials. Some of the dissolved organic materials are recalcitrant and persist in the ocean for a long time, thus becoming a reservoir of carbon storage in the ocean and making an impact on climate change [52]. 

The association of microorganisms and recalcitrant dissolved organic matter is not well understood. Since the biogeochemical interaction is highly complex, it is likely to involve multiple phases. Conceptual framework and hypotheses can be built on knowledge and data from systems biology. Microbial metabolomics serves as a crucial link for such understanding because recalcitrant carbon cycling is intimately connected with microbial processes [52]. 

### 3 Microbial Metabolism Reconstruction

This review concludes with an exciting prospective for systems biology – the reconstruction and analysis of microbial metabolism. A paradigm shift has taken place because of the knowledge gained in post genomic era. The unidirectional flow of biological information from genes to proteins is now being reevaluated. The central dogma of molecular biology accepted for decades is replaced by dynamic interactions (e.g., feedback loops) and multiple connections between metabolites in cellular processing [7]. This results from the recognition that metabolic networks are complex, consisting of biochemical reactions and associated molecular components such as enzymes, substrates, products, cofactors [22, 27, 91]. As mentioned previously, metabolomics complements transcriptomics and proteomics with added value. Generated from expressions and changes of systems biology components, metabolomes are situated more downstream of the hierarchical functional analyses. Therefore, it more accurately reflects microbial phenotypes. Elucidation of cellular networks requires inputs from metabolomics because metabolic fluxes and interactions cannot be calculated or deduced from transcripts and proteins alone [7]. 

Reconstruction of metabolic networks to analyze cellular processes can be conducted by large sets of “omics”-related data combined with a number of computational methods and tools [22, 57]. Indeed, metabolic network reconstructions and models at a genome scale have been accomplished with different microorganism, even for those with only scarce information in the literature [31, 57]. The systematic process of reconstructing a metabolic network generally commences with an annotated genome and concludes with a predictive model of microbial physiology [91]. All systems biology components have inputs into the reconstruction of metabolic pathways, and various networks help refine and expand the metabolic content [7, 27]. Procedures involved in this process utilize automation analysis coupled with manual curation to address gaps and reconcile known metabolic functions with genetic and biochemical data [31, 32]. 

Furthermore, recent advances in technologies enable metabolite profiling which adds another dimension to biochemical pathways [6, 22]. A complete inventory of metabolomes can now be achieved by high-throughput mass spectrometry with platforms capable of high accuracy resolution [7, 9, 38]. Thus, novel metabolites can be discovered and followed by metabolic correlation analysis to infer biochemical connectivity. The predicted network is further validated by phenotype experiments under different growth conditions [22, 32]. This integrated approach accommodates studies of metabolic pathways that are peripheral albeit important players in microbial physiology [31]. 

One practical application in this arena is metabolic engineering. Microorganisms are prime candidates for industrial production of desired products ranging from pollutant degradation to renewable energy [7, 22]. Rather than rationally altering an organism *via *genetic manipulations to achieve enhanced performance, a holistic understanding of different stages in the hierarchical organization (from genome to metabolome) enables biological systems to be designed and controlled more accurately [27]. Global information generated from the “omics” as well as pre-existing knowledge of microbial physiology are integrated by mathematical and statistical methods which in turn are used to build predictive models. Any disparities are resolved and the models are further refined by experimental observations in an iterative manner [22, 27]. This continuous and repetitious cycle of perturbation biology is key to the systems biology approach and it is especially effective in constructing models of metabolic networks and dynamic interactions between the biological components [26].

A wealth of annotation tools, software and databases greatly facilitate the reconstruction and model processes. Newly developed computational tool boxes continue to appear in the literature for consideration [22, 31, 32, 54, 92]. As a result, structured knowledge bases integrating vast amounts of data, databases and network reactions into one resource with standard nomenclature for comparison becomes a critical need for metabolic reconstruction and models. The establishment of the biochemical, genomic and genetic (BiGG) knowledge base serves as a metabolic model repository and satisfies such a need [22, 57]. Currently, BiGG makes available the reconstructions of genome scale metabolic networks from six organisms spanning three major branches of the Tree of Life. Among them are *E. coli* (a model organism), *Helicobacter pylori* (Gram negative bacterium), *Staphylococcus aureus* (Gram positive bacterium) and *Methanosarcina barkeri *(archaea)*. *This knowledge base fulfills one of the necessities in systems biology: the access to a curated collection of metabolic models and reconstructions operating within the Constraint Based Reconstruction and Analysis (COBRA) framework. Another resource that integrates metabolic data is the MetaCyc database containing highly curated small molecule metabolites [55]. Because the metabolic pathways and enzyme data in this database have been demonstrated experimentally, it serves as a reference to general metabolism. The MetaCychas collected more than 1,400 pathways from all domains of life [55]. Among the prokaryotes, the Proteobacteria led the number of pathways (750 in total).

The *de novo* reconstruction of metabolic maps by genome annotation and computational predictions worked well for a number of organisms. Nonetheless, there are limitations in addressing gaps. One major obstacle is the unknown sequences for enzymes involved in certain metabolic activities [7, 22]. These orphan reactions can be global in nature and account for up to 30-40% of the known metabolic activities [7, 32]. The BiGG knowledge base can provide part of the solution as shown in *E. coli *[57]. Another knowledge gap concerns the unknown metabolic reactions and/or pathways frequently missed by automatic reconstruction methods. Especially critical are those not essential for survival (such as those only expressed under specific environmental conditions) and thus tend to fall outside the scope of network reconstruction and analysis [32]. The integrated approach that incorporates metabolite profiling addresses some of these issues [6, 22].

Metabolic reconstruction can take another perspective and look beyond biochemical processes and networks in one organism for a more global overview of biochemical reactions involved in central metabolic pathways (such as carbon and nitrogen utilization). Deduced metabolism models for four intracellular pathogens are good examples in this regard [91]. In general, the carbon metabolism seemed to be flexible and allowing alternative substrates to be utilized. On the other hand, each organism showed unique adaptations in response to nutrient supplied by the host. These pathogen-specific adjustments may have a role in expressing virulence factors to accommodate its intracellular lifestyle. Another study conducted a system-based comparison of metabolism between related species [53]. Surveying the landscape of 19 genomes of the *Shewanella* genus, the researchers systematically mapped their carbohydrate utilization pathways. This “sugar catabolome” reconstruction allowed novel functional assignments of previously unknown (or less defined) components of transporters, regulators, and enzymes in different species. Additionally, 17 peripheral sugar catabolic pathways were elucidated and compared with the genomes to better understand the physiology and adaptation of *Shewanella* in specific environmental conditions [53]. It is foreseeable this type of “Genomic Encyclopedia” for a certain substrate utilization pathway can be reconstructed from different groups of microorganisms, thus expanding knowledge-based repositories to further the understanding of systems microbiology. 

The reconstruction and modeling of microbial metabolism are complicated processes that involve repetitive steps and expertise evaluations. This short synopsis is not intended to delineate various approaches, but to bring awareness of the great potential metabolomics exerts in this burgeoning field and leave the details to many good reviews available in the literature.

## SUMMARY

Selected websites useful for microbial metabolomics research are included in the following table. Most of them have been mentioned in this article. These are intended as representative of the vast amount of resources currently available to the scientific community. 

## CONCLUSION

The next decade of genomics will continue to emphasize function analyses and promote a systematic and integrated approach for life science studies. Microbiological research has already adopted this perspective, yielding results and insights not possible with traditional methodology. Microbes are no longer regarded as isolated organisms existing in a system, but rather an integrated component for understanding functional biology [32, 57]. 

In this review, selected topics in microbial metabolomics were discussed in relation to human and environmental ecosystems. Microbial metabolism has served as the foundation of biochemical pathway deduction for decades, and only recently was global reconstruction and evaluation of cellular processes made possible by informatics input from systems biology as mentioned in the last section. Although the focus was on microorganisms, prokaryotes constituted the majority of the discussion. Others, such as viromes, are not included because they are relatively understudied at this time. Nonetheless, their impacts on various ecosystems are increasingly being recognized [93]. 

As a relatively new discipline, microbial metabolomics is not without trials and setbacks. It is hoped that the knowledge gained from other “omics” will smooth the path forward. Indeed, challenges such as standardization, metabolic annotation, measurements of metabolite flux, dynamic range and depth-of-coverage, as well as large amounts of informatics and databases have been identified and solutions proposed in conferences, working groups and publications [7, 14, 22, 25, 33, 35, 94]. Efficient interdisciplinary collaboration is paramount to the advancement of systems biology. The Human Microbiome Project sets a good precedence, and the recent establishment of Systems Biology Program for Infectious Disease Research sponsored by National Institute of Allergy and Infectious Disease (NIAID) is another innovative paradigm to address obstacles in pathogen and host interaction research [26]. Societies of metabolomics with members representing different areas of expertise testify to the effectiveness of collaboration and sharing of knowledge. Since systems biology represents a paradigm shift and utilizes an integrated approach very different from traditional studies, it will take some time before moving from technology and computation-driven research to comprehensively understanding the data and their implications. Metabolomics research is now evident in academia, industry and government with more than 500 papers published on this subject annually. Hence, it is imminent that microbial metabolomics will soon join the rank and makes its mark in systems microbiology. 

## Figures and Tables

**Table 1:** A Few Representative Websites are Good Resources and Contain Useful Information for Microbial Metabolomics

**Metagenomics**
Metagenomics projects have served as foundation for systems microbiology. A few examples of microbial communities are listed here. Human Microbiome Project: https://commonfund.nih.gov/hmp/ NIAID Systems Biology Centers – currently focus on infectious diseases and pathogens (2 bacteria and 2 viruses):http://www.niaid.nih.gov/LabsAndResources/resources/dmid/sb/Pages/default.aspx DOE Joint Genome Institute Integrated Microbial Genomes with Microbiome Samples. http://img.jgi.doe.gov/cgi-bin/m/main.cgi DOE Joint Genome Institute Metagenomics Program-Exploration of Microbial Communities. http://genome.jgi-psf.org/programs/metagenomes/metagenomic-projects.jsf

**Metabolome Consortia**
Consortia have been established as repositories and databases for metabolic studies. The majority of current consortia are aimed at human metabolites; nonetheless, researchers can leverage these existing collaborative efforts to spearhead microbial metabolite profiling. Human Metabolome Project: http://www.metabolomics.ca/index.htm LIPID MAPS: http://www.lipidmaps.org/ Madison Metabolomics Consortium Database: http://mmcd.nmrfam.wisc.edu/ Consortium for Metabonomic Toxicology (COMET): http://bc-comet.sk.med.ic.ac.uk/

**Metabolic Reconstruction and Modeling**
Metabolic pathway reconstruction and modeling are multi-faceted which requires inputs from different databases and utilizes a variety of tools. The sites below are examples of knowledge base for the vast amount of technologies, tools, and reference databases. Additionally, Table **1** in Durot *et al.* paper contains a number of databases and tools [22]. BiGG knowledgebase: http://bigg.ucsd.edu MetaCyc database: http://MetaCyc.org Rainer Breitling’s homepage: http://gbic.biol.ru.gnl/-rbreitling Biomodels: http://www.ebi.ac.uk/biomodels/

**Societies**
Metabolomics Society: http://www.metabolomicssociety.org/ The Metabolomics Standards Initiative; http://msi-workgroups.sourceforge.net/

## References

[1] Alberts B (2011). Lessons from genomics. Science.

[2] Fleischmann R D, Adams M D, White O, Clayton R A, Kirkness E F, Kerlavage A R, Bult C J, Tomb J F, Dougherty B A, Merrick J M (1995). Whole-genome random sequencing and assembly of *Haemophilus influenzae* Rd. Science.

[3] NCBI Genome Project - Prokaryotic Genomes. http://www.ncbi. nlm.nih.gov/genomes/lproks.cgi.

[4] DOE Joint Genome Institute Metagenomcis Program-Exploration of Microbial Communities. http://genome.jgi-psf.org/programs/metagenomes/metagenomic-projects.jsf.

[5] Morrow K J (2010). Mass spec central to metabolomics. Genetic Engineering & Biotechnology News.

[6] Villas-Boas S G, Mas S, Akesson M, Smedsgaard J, Nielsen J (2005). Mass spectrometry in metabolome analysis. Mass Spectrom. Rev.

[7] Hollywood K, Brison D R, Goodacre R (2006). Metabolomics: current technologies and future trends. Proteomics.

[8] Bundy J G, Willey T L, Castell R S, Ellar D J, Brindle K M (2005). Discrimination of pathogenic clinical isolates and laboratory strains of *Bacillus cereus* by NMR-based metabolomic profiling. FEMS Microbiol. Lett.

[9] Garcia D E, Baidoo E E, Benke P I, Pingitore F, Tang Y J, Villa S, Keasling J D (2008). Separation and mass spectrometry in microbial metabolomics. Curr. Opin. Microbiol.

[10] Nicholson J K, Lindon J C (2008). Metabonomics. Nature.

[11] Ohdoi C, Nyhan W L, Kuhara T (2003). Chemical diagnosis of Lesch-Nyhan syndrome using gas chromatography-mass spectrometry detection. J. Chromatogr. B. Analyt. Technol. Biomed. Life Sci.

[12] Li M, Wang B, Zhang M, Rantalainen M, Wang S, Zhou H, Zhang Y, Shen J, Pang X, Wei H, Chen Y, Lu H, Zuo J, Su M, Qiu Y, Jia W, Xiao C, Smith L M, Yang S, Holmes E, Tang H, Zhao G, Nicholson J K, Li L, Zhao L (2008). Symbiotic gut microbes modulate human metabolic phenotypes. Proc. Natl. Acad. Sci. USA.

[13] Brindle J T, Antti H, Holmes E, Tranter G, Nicholson J K, Bethell H W, Clarke S, Schofield P M, McKilligin E, Mosedale D E, Grainger D J (2002). Rapid and noninvasive diagnosis of the presence and severity of coronary heart disease using 1H-NMR-based metabonomics. Nat. Med.

[14] Rochfort S (2005). Metabolomics reviewed: a new "omics" platform technology for systems biology and implications for natural products research. J. Nat. Prod.

[15] Seli E, Botros L, Sakkas D, Burns D H (2008). Noninvasive metabolomic profiling of embryo culture media using proton nuclear magnetic resonance correlates with reproductive potential of embryos in women undergoing *in vitro* fertilization. Fertil. Steril.

[16] Spratlin J L, Serkova N J, Eckhardt S G (2009). Clinical applications of metabolomics in oncology: a review. Clin. Cancer Res.

[17] Ketchum R E, Rithner C D, Qiu D, Kim Y S, Williams R M, Croteau R B (2003). *Taxus* metabolomics: methyl jasmonate preferentially induces production of taxoids oxygenated at C-13 in *Taxus x* media cell cultures. Phytochemistry.

[18] Fiehn O (2003). Metabolic networks of *Cucurbita maxima* phloem. Phytochemistry.

[19] Schaneberg B T, Crockett S, Bedir E, Khan I A (2003). The role of chemical fingerprinting: application to *Ephedra*. Phytochemistry.

[20] Burns J, Fraser P D, Bramley P M (2003). Identification and quantification of carotenoids, tocopherols and chlorophylls in commonly consumed fruits and vegetables. Phytochemistry.

[21] Stobiecki M, Matysiak-Kata I, Franski R, Skala J, Szopa J (2003). Monitoring changes in anthocyanin and steroid alkaloid glycoside content in lines of transgenic potato plants using liquid chromatography/mass spectrometry. Phytochemistry.

[22] Durot M, Bourguignon P Y, Schachter V (2009). Genome-scale models of bacterial metabolism: reconstruction and applications. FEMS Microbiol. Rev.

[23] Lindon J C, Keun H C, Ebbels T M, Pearce J M, Holmes E, Nicholson J K (2005). The Consortium for Metabonomic Toxicology (COMET): aims, activities and achievements. Pharmacogenomics.

[24] Wishart D S, Knox C, Guo A C, Eisner R, Young N, Gautam B, Hau D D, Psychogios N, Dong E, Bouatra S, Mandal R, Sinelnikov I, Xia J, Jia L, Cruz J A, Lim E, Sobsey C A, Shrivastava S, Huang P, Liu P, Fang L, Peng J, Fradette R, Cheng D, Tzur D, Clements M, Lewis A, De Souza A, Zuniga A, Dawe M, Xiong Y, Clive D, Greiner R, Nazyrova A, Shaykhutdinov R, Li L, Vogel H J, Forsythe I (2009). HMDB: a knowledgebase for the human metabolome. Nucleic Acids Res.

[25] Kyrpides N C (2009). Fifteen years of microbial genomics: meeting the challenges and fulfilling the dream. Nat. Biotechnol.

[26] Aderem A, Adkins J N, Ansong C, Galagan J, Kaiser S, Korth M J, Law G L, McDermott J G, Proll S C, Rosenberger C, Schoolnik G, Katze M G (2011). A systems biology approach to infectious disease research: innovating the pathogen-host research paradigm. MBio.

[27] Vemuri G N, Aristidou A A (2005). Metabolic engineering in the -omics era: elucidating and modulating regulatory networks. Microbiol. Mol. Biol. Rev.

[28] Ohashi Y, Hirayama A, Ishikawa T, Nakamura S, Shimizu K, Ueno Y, Tomita M, Soga T (2008). Depiction of metabolome changes in histidine-starved *Escherichia coli* by CE-TOF-MS. Mol. Biosyst.

[29] Gill S R, Pop M, Deboy R T, Eckburg P B, Turnbaugh P J, Samuel B S, Gordon J I, Relman D A, Fraser-Liggett C M, Nelson K E (2006). Metagenomic analysis of the human distal gut microbiome. Science.

[30] Forster J, Famili I, Fu P, Palsson B O, Nielsen J (2003). Genome-scale reconstruction of the *Saccharomyces cerevisiae* metabolic network. Genome Res.

[31] Feist A M, Herrgard M J, Thiele I, Reed J L, Palsson B O (2009). Reconstruction of biochemical networks in microorganisms. Nat. Rev. Microbiol.

[32] Breitling R, Vitkup D, Barrett M P (2008). New surveyor tools for charting microbial metabolic maps. Nat. Rev. Microbiol.

[33] Sintchenko V, Iredell J R, Gilbert G L (2007). Pathogen profiling for disease management and surveillance. Nat. Rev. Microbiol.

[34] Smith C A, O'Maille G, Want E J, Qin C, Trauger S A, Brandon T R, Custodio D E, Abagyan R, Siuzdak G (2005). METLIN: a metabolite mass spectral database. Ther. Drug Monit.

[35] Nicholson J K, Holmes E, Wilson I D (2005). Gut microorganisms, mammalian metabolism and personalized health care. Nat. Rev. Microbiol.

[36] VerBerkmoes N C, Denef V J, Hettich R L, Banfield J F (2009). Systems biology: Functional analysis of natural microbial consortia using community proteomics. Nat. Rev. Microbiol.

[37] Pusch W, Kostrzewa M (2005). Application of MALDI-TOF mass spectrometry in screening and diagnostic research. Curr. Pharm. Des.

[38] Dunn W B, Bailey N J, Johnson H E (2005). Measuring the metabolome: current analytical technologies. Analyst.

[39] Venter J C (2011). The human genome at 10: successes and challenges. Science.

[40] Ventura M, O'Flaherty S, Claesson M J, Turroni F, Klaenhammer T R, van Sinderen D, O'Toole P W (2009). Genome-scale analyses of health-promoting bacteria: probiogenomics. Nat. Rev. Microbiol.

[41] Torsvik V, Ovreas L (2002). Microbial diversity and function in soil: from genes to ecosystems. Curr. Opin. Microbiol.

[42] Tringe S G, von Mering C, Kobayashi A, Salamov A A, Chen K, Chang H W, Podar M, Short J M, Mathur E J, Detter J C, Bork P, Hugenholtz P, Rubin E M (2005). Comparative metagenomics of microbial communities. Science.

[43] Qin J, Li R, Raes J, Arumugam M, Burgdorf K S, Manichanh C, Nielsen T, Pons N, Levenez F, Yamada T, Mende D R, Li J, Xu J, Li S, Li D, Cao J, Wang B, Liang H, Zheng H, Xie Y, Tap J, Lepage P, Bertalan M, Batto J M, Hansen T, Le Paslier D, Linneberg A, Nielsen H B, Pelletier E, Renault P, Sicheritz-Ponten T, Turner K, Zhu H, Yu C, Jian M, Zhou Y, Li Y, Zhang X, Qin N, Yang H, Wang J, Brunak S, Dore J, Guarner F, Kristiansen K, Pedersen O, Parkhill J, Weissenbach J, Bork P, Ehrlich S D (2010). A human gut microbial gene catalogue established by metagenomic sequencing. Nature.

[44] DOE Joint Genome Institute Integrated Microbial Genomes with Microbiome Samples. http://img.jgi.doe.gov/cgi-bin/m/main.cgi.

[45] Rusch D B, Halpern A L, Sutton G, Heidelberg K B, Williamson S, Yooseph S, Wu D, Eisen J A, Hoffman J M, Remington K, Beeson K, Tran B, Smith H, Baden-Tillson H, Stewart C, Thorpe J, Freeman J, Andrews-Pfannkoch C, Venter J E, Li K, Kravitz S, Heidelberg J F, Utterback T, Rogers Y H, Falcon L I, Souza V, Bonilla-Rosso G, Eguiarte L E, Karl D M, Sathyendranath S, Platt T, Bermingham E, Gallardo V, Tamayo-Castillo G, Ferrari M R, Strausberg R L, Nealson K, Friedman R, Frazier M, Venter J C (2007). The Sorcerer II Global Ocean Sampling expedition: northwest Atlantic through eastern tropical Pacific. PLoS Biol.

[46] Walker A (2010). A glut from the gut: metagenomics takes a giant step forward. Nat. Rev. Microbiol.

[47] Tringe S G, Rubin E M (2005). Metagenomics: DNA sequencing of environmental samples. Nat. Rev. Genet.

[48] Vogel T M, Simonet P, Jansson J K, Hirsch P R, Tiedje J M, van Elsas J K, Bailey M J, Nalin R (2009). TerraGenome: a consortium for the sequencing of a soil metagenome. Nat. Rev. Microbiol.

[49] Turnbaugh P J, Ley R E, Mahowald M A, Magrini V, Mardis E R, Gordon J I (2006). An obesity-associated gut microbiome with increased capacity for energy harvest. Nature.

[50] Raes J, Bork P (2008). Molecular eco-systems biology: towards an understanding of community function. Nat. Rev. Microbiol.

[51] Raes J, Foerstner K U, Bork P (2007). Get the most out of your metagenome: computational analysis of environmental sequence data. Curr. Opin. Microbiol.

[52] Jiao N, Herndl G J, Hansell D A, Benner R, Kattner G, Wilhelm S W, Kirchman D L, Weinbauer M G, Luo T, Chen F, Azam F (2010). Microbial production of recalcitrant dissolved organic matter: long-term carbon storage in the global ocean. Nat. Rev. Microbiol.

[53] Rodionov D A, Yang C, Li X, Rodionova I A, Wang Y, Obraztsova A Y, Zagnitko O P, Overbeek R, Romine M F, Reed S, Fredrickson J K, Nealson K H, Osterman A L (2010). Genomic encyclopedia of sugar utilization pathways in the *Shewanella* genus. BMC Genomics.

[54] Thomas S, Bonchev D (2010). A survey of current software for network analysis in molecular biology. Hum. Genomics.

[55] Caspi R, Altman T, Dale J M, Dreher K, Fulcher C A, Gilham F, Kaipa P, Karthikeyan A S, Kothari A, Krummenacker M, Latendresse M, Mueller L A, Paley S, Popescu L, Pujar A, Shearer A G, Zhang P, Karp P D (2010). The MetaCyc database of metabolic pathways and enzymes and the BioCyc collection of pathway/genome databases. Nucleic Acids Res.

[56] Peterson J, Garges S, Giovanni M, McInnes P, Wang L, Schloss J A, Bonazzi V, McEwen J E, Wetterstrand K A, Deal C, Baker C C, Di Francesco V, Howcroft T K, Karp R W, Lunsford R D, Wellington C R, Belachew T, Wright M, Giblin C, David H, Mills M, Salomon R, Mullins C, Akolkar B, Begg L, Davis C, Grandison L, Humble M, Khalsa J, Little A R, Peavy H, Pontzer C, Portnoy M, Sayre M H, Starke-Reed P, Zakhari S, Read J, Watson B, Guyer M (2009). The NIH Human Microbiome Project. Genome Res.

[57] Schellenberger J, Park J O, Conrad T M, Palsson B O (2010). BiGG: a Biochemical Genetic and Genomic knowledgebase of large scale metabolic reconstructions. BMC Bioinformatics.

[58] Turnbaugh P J, Ley R E, Hamady M, Fraser-Liggett C M, Knight R, Gordon J I (2007). The human microbiome project. Nature.

[59] Pennisi E (2010). Body's hardworking microbes get some overdue respect. Science.

[60] Lemon K P, Klepac-Ceraj V, Schiffer H K, Brodie E L, Lynch S V, Kolter R (2010). Comparative analyses of the bacterial microbiota of the human nostril and oropharynx. MBio.

[61] Atarashi K, Tanoue T, Shima T, Imaoka A, Kuwahara T, Momose Y, Cheng G, Yamasaki S, Saito T, Ohba Y, Taniguchi T, Takeda K, Hori S, Ivanov II, Umesaki Y, Itoh K, Honda K (2011). Induction of colonic regulatory T cells by indigenous *Clostridium* species. Science.

[62] Grice E A, Segre J A (2011). The skin microbiome. Nat. Rev. Microbiol.

[63] Grice E A, Kong H H, Conlan S, Deming C B, Davis J, Young A C, Bouffard G G, Blakesley R W, Murray P R, Green E D, Turner M L, Segre J A (2009). Topographical and temporal diversity of the human skin microbiome. Science.

[64] Grice E A, Snitkin E S, Yockey L J, Bermudez D M, Liechty K W, Segre J A (2010). Longitudinal shift in diabetic wound microbiota correlates with prolonged skin defense response. Proc. Natl. Acad. Sci. USA.

[65] Kassinen A, Krogius-Kurikka L, Makivuokko H, Rinttila T, Paulin L, Corander J, Malinen E, Apajalahti J, Palva A (2007). The fecal microbiota of irritable bowel syndrome patients differs significantly from that of healthy subjects. Gastroenterology.

[66] Ley R E, Lozupone C A, Hamady M, Knight R, Gordon J I (2008). Worlds within worlds: evolution of the vertebrate gut microbiota. Nat. Rev. Microbiol.

[67] Rawls J F, Samuel B S, Gordon J I (2004). Gnotobiotic zebrafish reveal evolutionarily conserved responses to the gut microbiota. Proc. Natl. Acad. Sci. USA.

[68] Ley R E, Turnbaugh P J, Klein S, Gordon J I (2006). Microbial ecology: human gut microbes associated with obesity. Nature.

[69] Spor A, Koren O, Ley R (2011). Unravelling the effects of the environment and host genotype on the gut microbiome. Nat. Rev. Microbiol.

[70] Perry G H, Dominy N J, Claw K G, Lee A S, Fiegler H, Redon R, Werner J, Villanea F A, Mountain J L, Misra R, Carter N P, Lee C, Stone A C (2007). Diet and the evolution of human amylase gene copy number variation. Nat. Genet.

[71] Beja-Pereira A, Luikart G, England P R, Bradley D G, Jann O C, Bertorelle G, Chamberlain A T, Nunes T P, Metodiev S, Ferrand N, Erhardt G (2003). Gene-culture coevolution between cattle milk protein genes and human lactase genes. Nat. Genet.

[72] Pridmore R D, Berger B, Desiere F, Vilanova D, Barretto C, Pittet A C, Zwahlen M C, Rouvet M, Altermann E, Barrangou R, Mollet B, Mercenier A, Klaenhammer T, Arigoni F, Schell M A (2004). The genome sequence of the probiotic intestinal bacterium *Lactobacillus johnsonii* NCC 533. Proc. Natl. Acad. Sci. USA.

[73] Gordon J I, Hooper L V, McNevin M S, Wong M, Bry L (1997). Epithelial cell growth and differentiation. II. Promoting diversity in the intestine: conversations between the microflora, epithelium,and diffuse GALT. Am. J. Physiol.

[74] Barnes M J, Powrie F (2011). The gut's *Clostridium* cocktail. Science.

[75] King T S, Elia M, Hunter J O (1998). Abnormal colonic fermentation in irritable bowel syndrome. Lancet.

[76] Willing B P, Russell S L, Finlay B B (2011). Shifting the balance: antibiotic effects on host-microbiota mutualism. Nat. Rev. Microbiol.

[77] Manichanh C, Rigottier-Gois L, Bonnaud E, Gloux K, Pelletier E, Frangeul L, Nalin R, Jarrin C, Chardon P, Marteau P, Roca J, Dore J (2006). Reduced diversity of faecal microbiota in Crohn's disease revealed by a metagenomic approach. Gut.

[78] Imhoff A, Karpa K (2009). Is there a future for probiotics in preventing *Clostridium difficile*-associated disease and treatment of recurrent episodes?. Nutr. Clin. Pract.

[79] Rohde C L, Bartolini V, Jones N (2009). The use of probiotics in the prevention and treatment of antibiotic-associated diarrhea with special interest in Clostridium difficile-associated diarrhea. Nutr. Clin. Pract.

[80] Sherman P M, Ossa J C, Johnson-Henry K (2009). Unraveling mechanisms of action of probiotics. Nutr. Clin. Pract.

[81] Wilmes P, Bowen B P, Thomas B C, Mueller R S, Denef V J, Verberkmoes N C, Hettich R L, Northen T R, Banfield J F (2010). Metabolome-proteome differentiation coupled to microbial divergence. MBio.

[82] Tyson G W, Chapman J, Hugenholtz P, Allen E E, Ram R J, Richardson P M, Solovyev V V, Rubin E M, Rokhsar D S, Banfield J F (2004). Community structure and metabolism through reconstruction of microbial genomes from the environment. Nature.

[83] Whitman W B, Coleman D C, Wiebe W J (1998). Prokaryotes: the unseen majority. Proc. Natl. Acad. Sci. USA.

[84] Schleper C, Jurgens G, Jonuscheit M (2005). Genomic studies of uncultivated archaea. Nat. Rev. Microbiol.

[85] Stepanauskas R, Sieracki M E (2007). Matching phylogeny and metabolism in the uncultured marine bacteria, one cell at a time. Proc. Natl. Acad. Sci. USA.

[86] Keller L, Surette M G (2006). Communication in bacteria: an ecological and evolutionary perspective. Nat. Rev. Microbiol.

[87] Bassler B L, Losick R (2006). Bacterially speaking. Cell.

[88] Wingreen N S, Levin S A (2006). Cooperation among microorganisms. PLoS Biol.

[89] Kessler J D, Valentine D L, Redmond M C, Du M, Chan E W, Mendes S D, Quiroz E W, Villanueva C J, Shusta S S, Werra L M, Yvon-Lewis S A, Weber T C (2011). A persistent oxygen anomaly reveals the fate of spilled methane in the deep Gulf of Mexico. Science.

[90] Hazen T C, Dubinsky E A, DeSantis T Z, Andersen G L, Piceno Y M, Singh N, Jansson J K, Probst A, Borglin S E, Fortney J L, Stringfellow W T, Bill M, Conrad M E, Tom L M, Chavarria K L, Alusi T R, Lamendella R, Joyner D C, Spier C, Baelum J, Auer M, Zemla M L, Chakraborty R, Sonnenthal E L, D'Haeseleer P, Holman H Y, Osman S, Lu Z, Van Nostrand J D, Deng Y, Zhou J, Mason O U (2010). Deep-sea oil plume enriches indigenous oil-degrading bacteria. Science.

[91] Eisenreich W, Dandekar T, Heesemann J, Goebel W (2010). Carbon metabolism of intracellular bacterial pathogens and possible links to virulence. Nat. Rev. Microbiol.

[92] Karp P D, Paley S M, Krummenacker M, Latendresse M, Dale J M, Lee T J, Kaipa P, Gilham F, Spaulding A, Popescu L, Altman T, Paulsen I, Keseler I M, Caspi R (2010). Pathway Tools version 13.0: integrated software for
pathway/genome informatics and systems biology. Brief Bioinform.

[93] Pennisi E (2011). Going viral: exploring the role of viruses in our bodies. Science.

[94] Metabolomics Society The Metabolomics Standards Initiative. http://msi-workgroups.sourceforge.net/.

